# Prolonged Sleep Deprivation and Continuous Exercise: Effects on Melatonin, Tympanic Temperature, and Cognitive Function

**DOI:** 10.1155/2014/781863

**Published:** 2014-07-06

**Authors:** Greggory R. Davis, Corey E. Etheredge, Lena Marcus, David Bellar

**Affiliations:** School of Kinesiology, University of Louisiana at Lafayette, Lafayette, LA 70504, USA

## Abstract

The purpose of this study was to examine tympanic temperature, melatonin, and cognitive function during a 36-hour endurance event. Nine male and three female participants took part in a 36-hour sustained endurance event without sleep (*N* = 12, mean age = 31.8 ± 5.0 yrs). Participants were stopped for data collection at checkpoints throughout the 36-hour event. Tympanic temperature was assessed, a psychomotor vigilance test (PVT) was administered, and saliva samples were collected. Salivary melatonin was determined via immunoassay. During the 36 hours of competition, melatonin levels were negatively correlated with the day of the race (*r*s = −0.277, *P* = 0.039) and positively associated with nighttime (*r*s = 0.316, *P* = 0.021). Significant main effects of tympanic temperature (*P* < 0.001), day of the competition (*P* = 0.018), and a tympanic temperature ∗ day of competition interaction (*P* < 0.001) were used to predict minor lapses in attention. No associations between melatonin levels and cognitive function were observed (*P* > 0.05). During the event tympanic temperature declined and was associated with an increase in lapses in attention. With sustained endurance events becoming more popular future research is warranted to evaluate the physiological impact of participation.

## 1. Introduction

Ultra-endurance events, similar to extended military operations, are now making their way into the general population in the form of competitive sporting events. These events generally consist of prolonged exercise bouts, sleep deprivation, and continuously changing environmental conditions. Given the rapidly growing popularity of these ultra-endurance events, it is important to examine the physiological changes that occur under these conditions in order to provide effective training recommendations for both athletes and military personnel.

Due to the severe physical and mental demands of military operations, many studies examining sleep deprivation and prolonged exercise have come from military settings. While these studies do provide useful information to the general population, military personnel often carry excess weight (e.g., backpacks), unlike ultra-endurance athletes. Studies have shown that extended exercise while carrying excess weight could lead to significantly impaired cognitive processing [1, 2]. In addition, while sleep deprivation alone does not seem to significantly impair physical work capability, exercise compounded with sleep deprivation appears to increase vulnerability for negative mood disturbances and impaired reaction times [3–5]. Thus, when sleep deprivation is combined with excess physical exertion, cognitive function is negatively affected.

In addition to sleep deprivation, military personnel and ultra-endurance athletes can experience drastically different environments depending upon the location and time of year they choose to compete. While acute cold exposure had no major effects on the metabolism of sleep-deprived individuals, repeated cold exposure was shown to negatively affect cognitive function [6–9]. Not only does cold exposure impact physical and mental capabilities during sustained, sleep-deprived exercise, sleep deprivation itself disrupts coordination of fluctuations in individual thermoregulation, a process crucial to normal cognitive function [10–14]. Should environmental temperature significantly drop during exercise, individuals could also experience a reduction in core body temperature, potentially leading to a change in cognitive function.

Previous studies have found that restricted sleep, in the absence of physical exertion, compromises many components of cognitive processing, alertness, and performance [15–22]. It is associated with a decline in core temperature and a concomitant decline in cognitive function [10–12] and a rise in the hormone melatonin, which governs the body's sleep-wake cycles and protects against sleep deprivation-induced behavioral and biochemical alterations [23]. Elevated levels of melatonin can also lead to the aforementioned impairment in cognitive function, as well as psychomotor vigilance [24, 25]. Furthermore, melatonin is known to increase long duration exercise, in which the body continuously works through the normal period of nighttime sleep, leading to altered circadian rhythms [26]. Taken together, these findings suggest that cognitive function, core temperature, and melatonin production all become altered during sleep deprivation in combination with prolonged exercise. Thus, the purpose of the present investigation was to examine the impact of a 36-hour ultra-endurance event on cognitive function, melatonin, and tympanic temperature among a group of competitors. We hypothesized that the combined effects of sleep deprivation and prolonged exercise would cause a drop in core temperature and cognitive function, while melatonin levels would rise throughout the event.

## 2. Methods

### 2.1. Subjects

The subjects consisted of 9 male and 3 female participants who took part in a 36-hour sustained endurance without sleep (*N* = 12, mean age = 31.8 ± 5.0 yrs). All subjects gave written informed consent prior to participating in the data collection. The participants completed a Leisure Physical Activity Survey [1] prior to the event (Total Aerobic Exercise Score = 7.1 ± 1.8, Mean Total Weightlifting Score = 6.7 ± 1.9) with results suggesting high levels of physical activity.

### 2.2. Ultra-Endurance Event

The event was held in rural Illinois (Cuba, IL, USA) during the late summer. The event was 36 hours in duration and consisted of a variety of different tasks. Tasks included extended bucket carries (buckets approximately 22.5 kg each for men and 15 kg each for women), extended marches with heavy packs, weightlifting, swimming, body weight exercises, and running in a predetermined order that remained unknown to the participants. Each task was completed as quickly as possible by each participant and, following the completion of each task, participants returned to the starting tent to receive the next task assignment from race officials. All participants completed the same tasks in the same order, which minimized the variation in environmental conditions and required physical exertion. Each task required several hours to complete and some participants completed tasks several minutes faster than others. The participants were allowed rest breaks at the conclusion of each task; however, they were disqualified if they slept. Specific rest times varied between individuals, tasks, and total elapsed time. Participants had shorter rest times (approximately 10 minutes) early in the event and following lower-intensity tasks, whereas participants had longer rest times later in the event and following higher-intensity tasks (approximately 30 minutes). The order of finish was determined by the amount of work performed during the 36 hours. The average temperature, barometric pressure, and humidity during the competition were 28.1°C, 739.6 mmHg, and 75.5% RH, respectively. The average daytime temperature was 31.8°C. Research personnel remained at the starting tent for the duration of the event and collected data from participants between tasks, thus data were collected at five time-points throughout the 36-hour event.

### 2.3. Tympanic Temperature

After resting for at least ten minutes, body temperature was assessed via a tympanic thermometer (Genius 2 Tympanic thermometer, Covidien LLC, Mansfield, MA). The participant was seated in a chair and the researcher guided the tip of the thermometer into the ear canal in such a manner as to follow the natural anatomy.

### 2.4. Saliva Collection and Analysis

One-milliliter samples of whole unstimulated saliva were collected using a saliva collection aid and frozen on dry ice at the event location. Upon arrival at the lab, samples were transferred to a −30°C lab freezer until analysis. Melatonin levels were analyzed using a commercial ELISA assay kit (Salimetrics LLC, State College, PA). The intra-assay coefficient of variation was 2.75%.

### 2.5. Psychomotor Vigilance Testing

In order to quantify the effects the ultra-endurance event on cognitive function, a psychomotor vigilance test (PVT) was administered to the participants after resting for at least ten minutes. In an effort to avoid the potential of practice effects occurring during subsequent testing, participants were familiarized and practiced the test after giving informed consent. The PVT is a test of simple visual reaction time and was developed at the Walter Reed Army Institute of Research [16, 17]. The PVT was used to assess mean reaction time over a 5-minute time course. The test used random periods of time in which a target stimulus was displayed on the screen of a Palm handheld device. The program was set to display approximately 100 stimuli in the 300-second (5 minutes) period at randomly spaced intervals [17]. This program computed a mean reaction time to each stimulus. Both right-handed and left-handed individuals were accommodated.

### 2.6. Statistical Analysis

Data were analyzed for relationships with the day of the race (days 1, 2, and 3) and light/dark via nonparametric correlations. This analysis was conducted both for tympanic temperature measurements and for melatonin concentrations. Generalized linear modeling analysis was used to examine the relationship between minor lapses in attention (>500 ms in duration, but less than 1000 ms) and tympanic temperature and melatonin with day of the competition included in the model. All statistical analyses were conducted with a modern computerized statistical software package (JMP 11.0). Statistical significance was set a priori at alpha <0.05.

## 3. Results

### 3.1. Tympanic Temperature

**Figure 1:**
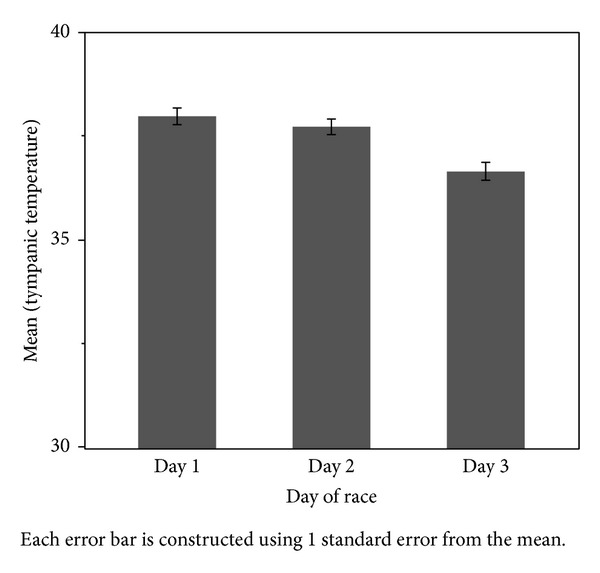
Mean tympanic temperature by day of competition. Error bars represent ± 1 SEM.

Over the course of the 36-hour event, there was a significant correlation between tympanic temperature and day of race (*r* = −0.444, *P* = 0.001; see Figure 1) with steady tympanic temperature decline (day 1: 38.0 ± 0.7°C, day 2: 37.7 ± 1.0°C, and day 3: 36.6 ± 0.7°C) during the event. Analysis also revealed a significant correlation between tympanic temperature and the light/dark cycle (*r*s = − 0.612, *P* = 0.000) (Mean Light Condition = 38.1 ± 0.86, Mean Dark Condition = 37.1 ± 0.86).

### 3.2. Melatonin

**Figure 2:**
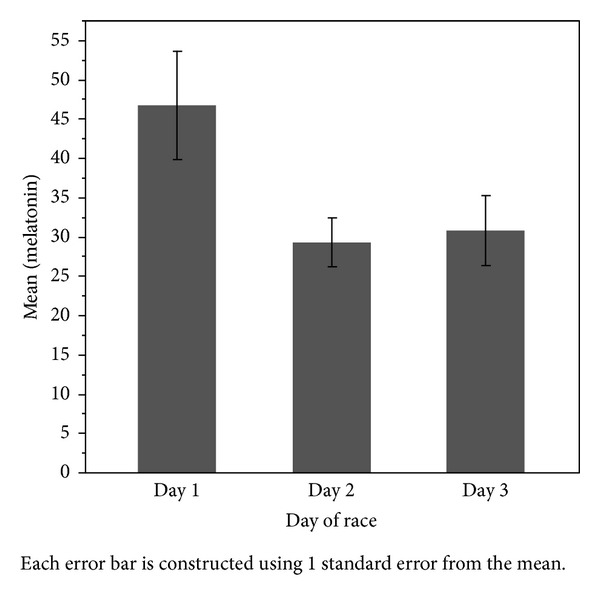
Mean melatonin by day of competition. Error bars represent ± SEM.

During the 36 hours of competition melatonin levels were negatively correlated with the day of the race (*r*s = − 0.277, *P* = 0.039; day 1: 46.8 ± 21.9 pg/mL, day 2: 29.3 ± 14.5 pg/mL, and day 3: 30.8 ± 12.6 pg/mL; see Figure 2) and positively associated with nighttime (*r*s = 0.316, *P* = 0.021; night: 40.5 ± 16.8 pg/mL, day: 24.2 ± 14.1 pg/mL).

### 3.3. Cognitive Function

**Figure 3:**
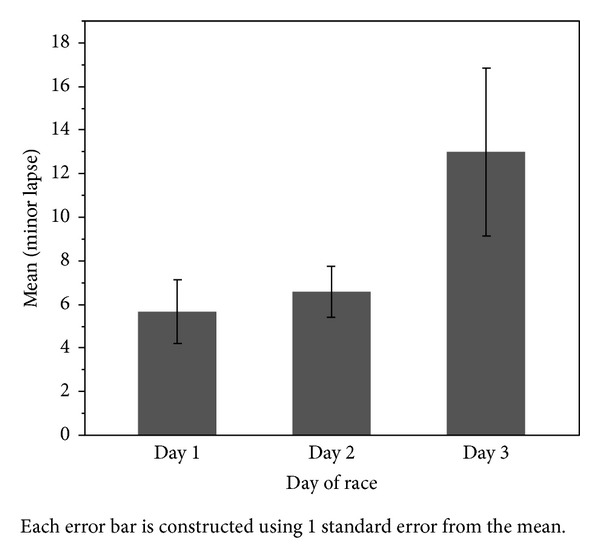
Mean major lapses by day of race. Error bars represent ± SEM.

General Linear Model analysis was conducted to predict minor lapses in attention (Omnibus Test *P* < 0.001) and resulted in significant main effects for tympanic temperature (*P* < 0.001), day of the competition (*P* = 0.018), and a tympanic temperature ∗ day of competition interaction (*P* < 0.001). During the 36 hours of the endurance event the participants' tympanic temperature declined (day 1: 38.0 ± 0.7°C, day 2: 37.7 ± 1.0°C, and day 3: 36.6 ± 0.7°C) and was associated with an increase in lapses in attention (day 1: 5.7 ± 4.3 lapses, day 2: 6.6 ± 5.7 lapses, and day 3: 13.0 ± 7.5 lapses). Major lapses by day of the competition can be seen in Figure 3.

## 4. Discussion

The decline in cognitive function associated with sleep deprivation has been well established. The current study is in agreement with previous research findings and further indicates that cognitive function may be dependent on tympanic temperature. The primary outcomes of the current study suggest that consecutive days of sleep deprivation result in an overall significant decline in cognitive function. In addition, the significant decrease in tympanic temperature throughout the 36-hour race despite the absence of a significant increase in salivary melatonin suggests that cognitive function may be directly affected by tympanic temperature, independent of melatonin concentration. These findings are supported by a previous research study which demonstrated that lower temperatures in the brain are associated with a greater number of lapses on the PVT during dark hours and that body temperature directly regulates cognitive function, independent of circadian rhythm [12]. Additional research, however, has indicated that decreased time in rapid eye movement sleep cycles (deep sleep) and increased time in wake cycles are associated with changes in core temperature and cognitive function [27]. Thus, altered sleep cycles, which are known to affect circadian patterns, likely affect cognitive function via direct changes in core temperature, rather than direct changes in melatonin production. The results of the current study further reinforce this notion.

Salivary melatonin concentrations followed typical light/dark oscillations throughout the race, yet melatonin concentrations were not higher during the second dark cycle versus the first dark cycle despite the prolonged sleep deprivation and drop in tympanic temperature. Previous sleep deprivation studies have shown that melatonin release increases with sleep deprivation [28, 29] and that the amplitude of melatonin release is dependent upon the duration of sleep deprivation [30], with longer sleep deprivation resulting in greater melatonin release and a greater drop in body temperature. This response to sleep deprivation is likely a physiological safeguard that encourages humans to sleep in order to regain normal physiological function. The current literature offers some potential insight as to why the current study did not demonstrate a significant change in melatonin amplitude despite a drop in tympanic temperature and prolonged sleep deprivation.

The most likely cause for the lack of change in melatonin observed in the current study was due to the physical exercise stimulus. Exercise at night has been shown to significantly blunt normal melatonin responses to dark cycles [31]. Thus, although melatonin concentrations were significantly higher during the dark cycles versus the light cycles, it is likely that the continuous stimulus of exercise was enough to attenuate an expected rise in melatonin during the second dark cycle of the 36-hour race. Physical exercise, however, did not alter the sleep-deprivation-associated drop in core temperature and cognitive function, further suggesting that sleep deprivation directly alters cognitive function via decreases in core temperature.

In addition to physical stress, many circadian rhythms are known to be affected by light and temperature and, therefore, it is also possible that the elevated environmental temperature in combination with the stressful conditions may have compromised the expected increase in melatonin release during the second dark cycle. It is well documented that core temperature is dependent upon environmental temperature, relative humidity, level of physical activity, and melatonin release [32, 33]. It is important to note that the environmental conditions for the current study were not controlled. At the peak environmental temperature of 36°C, the average tympanic temperature was 38.2°C; at the lowest environmental temperature of 21°C, the average tympanic temperature was 36.6°C. The coolest environmental temperature occurred at the end of the 36-hour race during the second dark cycle, so the possibility that sleep deprivation was responsible for the drop in tympanic temperature cannot be ruled out. However, melatonin levels were not significantly elevated during the second dark cycle when compared to the first dark cycle. Furthermore, tympanic temperature during the first dark cycle of the race, when participants were well-rested, averaged 38°C, versus 36.6°C during the second dark cycle, while the environmental temperature during the first dark cycle averaged 26.6°C, versus 21°C during the second dark cycle. These results indicate that although the participants were exerting themselves physically and mentally throughout the 36-hour period, tympanic temperature appeared to be more dependent upon the environment, rather than physical activity or melatonin levels. Tympanic temperature did change with the light/dark cycles and subsequent changes in melatonin concentration, with higher tympanic temperatures associated with lower salivary melatonin levels during light hours. However, environmental temperature was higher during light hours as well and melatonin concentration was not significantly altered during the second dark cycle yet tympanic temperature was significantly lower. This further reinforces the notion that tympanic temperature was more dependent on the environment than light/dark cycles or melatonin concentration. However, it cannot be ruled out that prolonged sleep deprivation played a major role in the decline in tympanic core temperature as well. It is worth noting that while rectal temperature provides the most accurate reflection of core temperature, such measurement techniques were not feasible given the nature of the current study. In addition, tympanic temperature measurements have been shown to be highly reliable [34], and since changes in core temperature were a primary outcome variable, rather than absolute core temperature values, tympanic measurements were appropriate.

While several sleep deprivation studies have been completed, this is the first study, to our knowledge, to examine the effects of 36 hours of continuous exercise. This type of activity is not common among the general public but may be more frequently observed in military training or missions as well as long-duration ultra-endurance races. Therefore, the findings presented here may prove particularly useful for groups of people that may undergo several hours or days of strenuous activity with minimal or no sleep time.

## 5. Conclusions

Clearly, the addition of exercise to prolonged sleep deprivation is effective at blunting melatonin responses to dark cycles, which may minimize the urge to sleep. However, decreased core temperature during prolonged activity and sleep deprivation was associated with compromised cognitive function demonstrating a need for the sleep to maintain cognitive function. Whether or not short sleep cycles (i.e., 1-2 hours) are effective enough to offset the drop in tympanic temperature and cognitive function associated with sleep deprivation is yet to be established but could prove useful, especially during prolonged activity.
